# Supplementary material for: A rule-based simulation model illuminates the role of asymmetric mitochondrial fission on beta-cell health
Source: NPJ Syst Biol Appl. 2026 May 5;12:64. doi: 10.1038/s41540-026-00732-0 (PMC13144372; doi:10.1038/s41540-026-00732-0)
Supplement: Supplementary file 1 — Supplementary Information [file 41540_2026_732_MOESM1_ESM.pdf]

Supplementary Information to: A Rule-Based Simulation Model Illuminates the Role of Asymmetric Mitochondrial Fission on Beta-Cell Health

Philipp Henning[1,*], Julia Schultz[2], Simone Baltrusch[2], Adelinde M. Uhrmacher[1]

1 Institute for Visual and Analytic Computing, University of Rostock, Rostock, Germany
2 Institute of Medical Biochemistry and Molecular Biology, Rostock University Medical Center, Rostock, Germany

* philipp.henning@uni-rostock.de

1 Submodels

In this section, the different submodels are described in detail. The literature used to derive the rules, rates, and rate parameters is presented. Some parameters in the literature are normalized to the cell volume or protein mass. In order to convert them to particle numbers, a beta-cell volume of 1025 fL [1] and a protein content of 0.25 mg protein per 10^6 cells [2]. Both values were measured for Min6 cells. All (sub)models and scripts for parameter estimation experiments can be found in the git repository `https://git.informatik.uni-rostock.de/phenning/beta-cell-models`.

1.1 Fission-Fusion Model

The fission-fusion submodel focuses on the dynamics of mitochondrial fission and fusion. It is used to estimate the parameters of the binding and unbinding of Drp1 to an anchor site, as well as the fission rate. Fig. S1 shows a schematic representation of the model. To reduce the computational cost of the simulation, Drp1 is clustered into bundles of 10 particles.

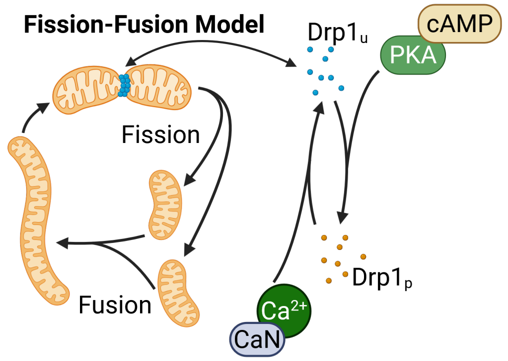

Fig. S1. Schematic representation of the fission-fusion model. Unphosphorylated $Drp1_{10}$ clusters (blue dots) bind to and dissociate from anchor sites on the mitochondrial membrane. When a sufficient amount of Drp1_10 accumulates on an anchor site, a fission event can occur. Mitochondria can also undergo fusion, establishing a continuous fission-fusion cycle that constantly remodels the mitochondrial network. Drp1 is phosphorylated by PKA, which is activated by cAMP, and dephosphorylated by CaN, activated by Ca^{2+}. Created in BioRender. Henning, P. (2026) `https://BioRender.com/77bgd7a`

1.1.1 Species

Species	Description
Cell	beta-cell
Mito(rand1:float*, rand2:float*)	Mitochondrion, rand1/2: used for fission
Mito_vol	volume particle $\widehat{=}$ 0.01 μm^3
Drp1_10(p:bool)	10 Drp1 proteins, p: phosphorization state
Anchor	anchor site for Drp1
PKA	cAMP-dependent protein kinase
cAMP	cyclic AMP
CaN	Calcineurin
Ca_100	100 Ca^{2+}

Table S1. Species used in the fission-fusion model.

1.1.2 Rules

Drp1 binding
The Dynamin-related protein 1 (Drp1) is a key factor in the mitochondrial fission dynamics. It can oligomerize into rings around a mitochondrion, as long as it is unphosphorylated [3], and constrict to initiate a fission event. However, Drp1 alone cannot bind to the outer membrane of the mitochondria but needs an anchor protein. It was shown that binding of Drp1 to the anchor protein is not the limiting factor of mitochondrial fission, but the oligomerization of Drp1 [4]. Therefore, we only model the oligomerization step of Drp1 binding, assuming that prior binding to the anchor protein has already occurred and does not limit the reaction rate.

```
Mito{Anchor} + Drp1_10(p==false) -> Mito{Anchor{Drp1_10}} @ kon/vol;
```
(S1)

Drp1 unbinding
Bound Drp1 can dissociate from anchor sites, following an isodesmic assembly model [5,6].

```
Mito{Anchor:a{Drp1_10}} -> Mito{Anchor} + Drp1_10 @@ 1/10*koff*#a;
```
(S2)

Drp1 phosphorylation
The phosphorylation of Drp1 is catalyzed by the cAMP-dependent protein kinase (PKA), which is activated by cAMP [3]. Reaction kinetics follow Michaelis-Menten dynamics with a Hill-function-like activation by cAMP [7,8].

```
Cell{Drp1_10(p==false):d + PKA:p} -> Cell{Drp1_10(p=true) + PKA}
 @@ 1/10*kcatpka*#p*#d*10/(KMpka*vol*NA+#d*10)*100*##(cAMP_100 in Cell)
 /(K_act_PKA+100*##(cAMP_100 in Cell));
```
(S3)

Drp1 dephosphorylation
Dephosphorylation of Drp1 is catalyzed by Calcineurin (CaN) [3], which is activated by Ca^{2+}. As for PKA, the kinetics follow Michaelis-Menton dynamics [9] with a Hill-function-like activation by Ca^{2+} [10].

```
Cell{Drp1_10(p==true):d + CaN:c} -> Cell{Drp1_10(p=false) + CaN}
 @@ 1/10*kcatCaN*#c*#d*10/(KMCaN*vol*NA+#d*10)*100*##(Ca_100 in Cell)
 /(K_act_CaN+100*##(Ca_100 in Cell));
```
(S4)

Mitochondrial fission

A fission event can occur once sufficient Drp1 has accumulated at an anchor site [5]. Mitochondria can undergo two different types of fission. The first is the midzone-fission event, where two roughly equal sister mitochondria are formed [11]. The fission is executed in two steps. First, the fission position is randomly generated (and saved in `rand1`) if a sufficient amount of Drp1 has accumulated in an anchor site and the Drp1 bound in this site returns to the cell compartment. The rate of the fission event depends on the size of the mitochondria, with larger ones being more likely to undergo fission [12, 13].

```
Mito:m{Anchor{Drp1_10:drp + ?solA}} ->
 Mito(rand1 = RandomGauss(0.5, 0.075)){Anchor} + Drp1_10+?solA     (S5)
 @@ if (#drp>N_fission) then k_fission*(#(Mito_vol in m))^3;
```

Immediately after the position of the fission event is determined, the fission is executed, and the content of the mother mitochondrion is distributed between the two sister mitochondria based on the fission position rand1.

```
Mito(rand1>0):m{?sol1[Mito.rand1] + ?sol2[1-Mito.rand1]} ->
 Mito(rand1=-1, rand2=-1){?sol1} + Mito(rand1=-1, rand2=-1){?sol2}  (S6)
 @@ immediately;
```

Equivalent rules govern the second type of fission events, peripheral fission. Here, the mitochondrion is divided into a small and a large sister mitochondrion. The position of the fission is stored in `rand2`.

```
Mito:m{Anchor{Drp1_10:drp + ?solA}} ->
 Mito(rand2 = RandomGauss(0.2, 0.04)){Anchor} + Drp1_10+?solA     (S7)
 @@ if (#drp>N_fission) then k_fission*(#(Mito_vol in m))^3;
```

```
Mito(rand2>0):m{?sol1[Mito.rand2] + ?sol2[1-Mito.rand2]} ->
 Mito(rand1=-1, rand2=-1){?sol1} + Mito(rand1=-1, rand2=-1){?sol2}  (S8)
 @@ immediately;
```

Mitochondrial fusion

The fusion of mitochondria is modeled as a simple rule with a constant reaction rate. In contrast to the fission rate, the fusion rate does not depend on the size of mitochondria [12].

```
Mito:m1{?solm1} + Mito:m2{?solm2} ->
 Mito(rand1=-1, rand2=-1){?solm1 + ?solm2} @@ k_fusion;           (S9)
```

1.1.3 Initial conditions

Species	Number	Ref.
`Cell`	1	-
$\texttt{Mito}(\texttt{rand1} = -1, \texttt{rand2} = -1)$	68	[14]
`Mito_vol`	100 per `Mito`	[14]
$\texttt{Drp1\_10}(\texttt{p} = \texttt{true})$	10000	[15]
$\texttt{Drp1\_10}(\texttt{p} = \texttt{false})$	10000	[15]
`Anchor`	612	[16, 17]
`PKA`	300000	[15]
`cAMP`	10000	[18]
`CaN`	350000	[15]
`Ca_100`	500	[19]

Table S2. Initialization of the fission-fusion submodel.

1.1.4 Parameters

Parameter	Description	Value	Ref.
`kon`	Binding rate or Drp1	$0.025\ \mu m^3/s$	fit to [5, 12, 14, 20]
`Kd`	Equilibrium constant for Drp1 binding	$0.301\ \mu mol/s$	fit to [5, 12, 14, 20]
`koff`	Unbinding rate of Drp1	$\texttt{kon} * \texttt{Kd}$	-
`k_fission`	Rate coefficient for fission	$8.211 \cdot 10^{-8} Hz$	fit to [5, 12, 14, 20]
`N_fission`	Number of Drp1 needed for fission	11	[5]
`rand1`	Position of midzone fission	N(0.5, 0.075)	[11]
`rand2`	Position of peripheral fission	N(0.2, 0.004)	[11]
`k_fusion`	Rate coefficient for fusion	$5.41 \cdot 10^{-4}$ 1/min	[12, 20]
`vol`	Volume of a Min6 cell	$1025\ \mu m^3$	[1]
`kcatCaN`	Rate constant for CaN	$7.6\ 1/s$	[9]
`KMCaN`	Michaelis constant for CaN	160 mM	[9]
`K_act_CaN`	Activation constant of CaN	14 nM	[10]
`kcatpka`	Rate constant ok PKA	4 1/s	[21]
`KMpka`	Michaelis constant for PKA	70 mM	[21]
`K_act_PKA`	Activation constant of PKA	$0.9\ \mu M$	[7]
`NA`	Avogadro constant	6.022 1/mol	-

Table S3. Parameters used in the fission-fusion submodel.

1.1.5 Parameter Estimation

The submodel was used to estimate the rate coefficient for Drp1 binding (`kon`), the equilibrium constant of Drp1 binding (`Kd`), and the rate coefficient for mitochondrial fission (`k_fission`). PySwarms [22] was used to minimize the relative quadratic difference

$$error = \sum_i \left(\frac{sim_i - lab_i}{lab_i} \right)^2 \tag{S10}$$

where sim_i represents the simulated model output and lab_i the corresponding experimental (wet-lab) data. The calibration targeted three experimental observables:

(i) the Drp1 assembly kinetics (2.6 s^{-1}) [5], (ii) the mitochondrial fission frequency (0.04 events min^{-1} $mitochondrion^{-1}$) [12, 20], and (iii) the average number of mitochondria in a MIN6 cell (68 mitochondria) [14]. The resulting parameter values are listed in Table S3, together with parameters directly taken from the literature.

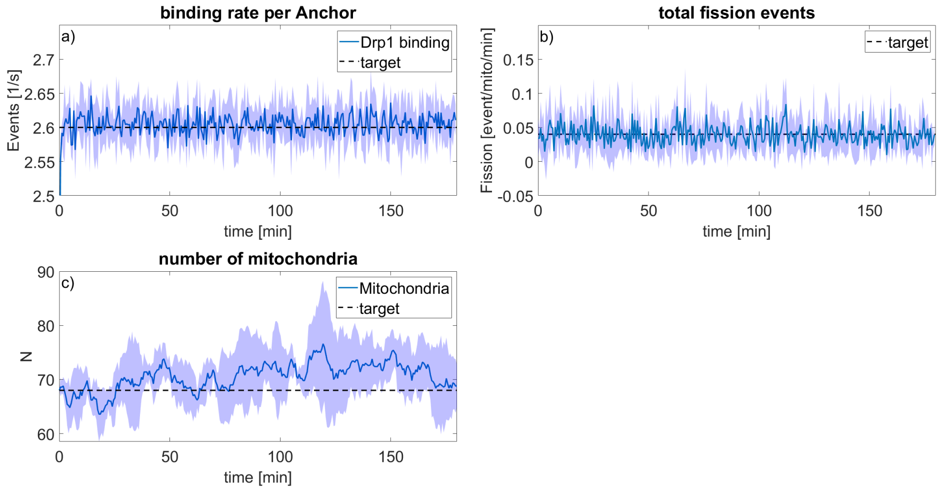

Fig. S2. Comparison between literature data and simulation results for a) the Drp1 binding rate [5], b) the mitochondrial fission rate [12, 20], and c) the number of mitochondria in a Min6 cell [14]. The shaded area shows the standard deviation of N=5 simulation runs.

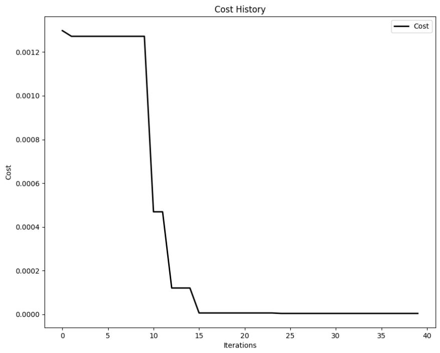

Fig. S3. Cost function of the pySwarms run to estimate the parameters of the fission-fusion model.

The simulation results of the fission-fusion submodel are shown in Fig. S2. After the parameter estimation, the submodel successfully reproduces the experimental target values for the Drp1 binding rate, mitochondrial fission rate, and the number of mitochondria. Fig. S3 shows the evolution of the error function during optimization. After approximately 15 iterations, the error converged, and an optimal parameter set was obtained.

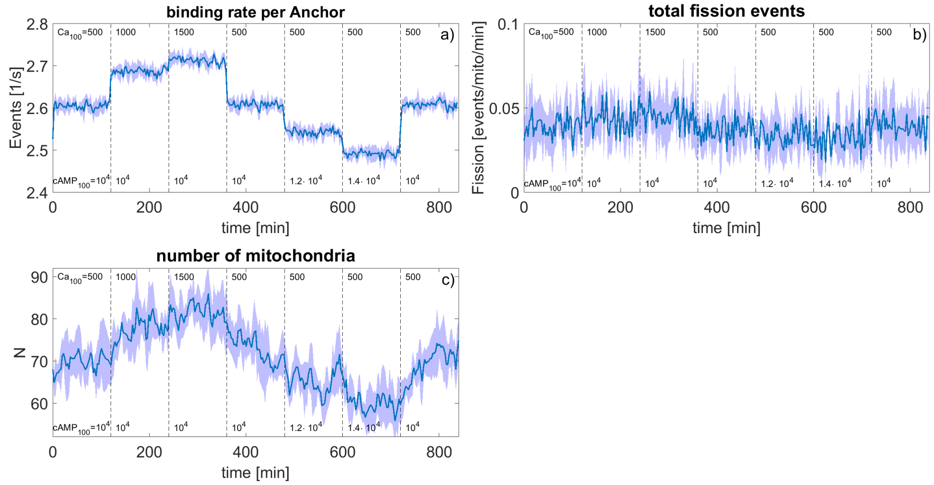

Fig. S4. Ca_{100} and $cAMP_{100}$ were varied during the runtime. The levels are noted at the top and bottom of the figures. a) shows the Drp1 binding rate, b) the mitochondrial fission rate, and c) the number of mitochondria in a Min6 cell. The shaded area shows the standard deviation of N=5 simulation runs.

1.1.6 Results

After calibration, the submodel was used to demonstrate the effects of calcium and cAMP on fission-fusion dynamics. In the experiments shown in Fig. S4, the system is initialized with 500 Ca_{100} and 10000 $cAMP_{100}$ particles as was the case in the experiments in Fig. S2. At the 2- and 4-hour marks, 500 Ca_{100} were added and removed at the 6-hour mark. cAMP was increased by 2000 particles at the 8- and 10-hour marks and returned to initial levels at t=12 hours. Calcium and cAMP vary the amount of (un)phosphorylated Drp1 by changing the activity of CaN and PKA (see Fig. S1). An increase in calcium leads to more unphosphorylated Drp1, which leads to more frequent binding to anchors (Fig. S4 a), a slightly higher fission rate (Fig. S4 b), and consequently an increased number of mitochondria. As cAMP stimulated Drp1 phosphorylation, it has the opposite effect.

1.2 ATP-Insulin secretion model

The ATP-Insulin submodel focuses on ATP production and consumption, calcium signaling, and insulin secretion, as shown in Fig. S5. It is used to estimate parameters for the ATP dynamic and the insulin secretion dynamic.

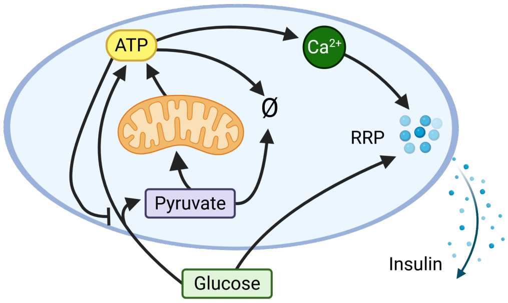

Fig. S5. Schematic representation of the ATP-Insulin secretion model. Glucose is metabolized to ATP through glycolysis and oxidative phosphorylation. The increase in ATP triggers an influx of Ca^{2+}, which induces insulin release from the rapid release pool (RRP). Replenishment of the RRP is regulated by the intracellular glucose concentration. Created in BioRender. Henning, P. (2026) `https://BioRender.com/ifjt5zy`

1.2.1 Species

Species	Description
Cell(Gluc: float[mol/L])	beta-cell, Gluc: glucose level
Mito	Mitochondrion
pyruvat_1000000	10^6 pyruvate molecules
ATP_1000000	10^6 ATP molecules
RRP_100	100 insulin molecules in the rapid releasable pool (RRP)
Ins_100	100 insulin molecules
Ca_100	100 Ca^{2+} atoms

Table S4. Species used in the ATP-Insulin secretion model.

1.2.2 Rules

Glycolysis
For glycolysis, it is assumed that there is a constant supply of glucose, that metabolic activity does not lower the glucose level, and that glucose shuttling into the cell is not a bottleneck of the metabolism. Consequently, glucose is modeled as an attribute of the cell, rather than a species, and the glucose shuttling is not implemented. The ten-step glycolytic pathway is simplified to a single reaction that produces two pyruvate molecules and two ATP molecules, with ATP acting as a feedback inhibitor. NADH is not modeled as a species; however, for subsequent reactions, it is assumed that one NADH is produced per pyruvate molecule.

```
Cell{ATP_1000000:atp} -> Cell{2 pyruvat_1000000 + 3 ATP_1000000}
 @@ Vm_glyc*Cell.Gluc/((Km_glyc+Cell.Gluc)*(1+#atp/Ki_glyc));
```
(S11)

This reaction is inhibited by ATP, consistent with regulatory control of key glycolytic enzymes [23].

Anaerobic fermentation
Pyruvate can be converted to lactate via anaerobic fermentation, consuming one NADH per pyruvate and thereby balancing the NADH generated in glycolysis.

```
pyruvat_1000000:pyr -> @@ Vmax_LDH*#pyr*1e6/(#pyr*1e6+Km_LDH_pyr);
```
(S12)

TCA cycle + oxidative phosphorylation
Alternatively, pyruvate is oxidized in the tricarboxylic acid cycle and oxidative phosphorylation (oxPhos) to produce ATP within mitochondria.

```
Mito + pyruvat_1000000:pyr -> Mito + 15 ATP_1000000
 @@ (1/75)*Vmax_PDH*#pyr*1e6/(#pyr*1e6+Km_PDH_pyr);
```
(S13)

In this reaction, NADH generated by glycolysis contributes to ATP production. The simplified pathway yields two ATP per glucose through glycolysis and fermentation, or 32 ATP per glucose through glycolysis and oxidative phosphorylation.
ATP consumption
ATP is consumed in various cellular processes in an ATP-dependent manner.

```
ATP_1000000:atp -> @@ k_ATP_u*#atp;
```
(S14)

Ca^{2+} dynamic
An increase in ATP concentration induces an influx of Ca^{2+} ions. This process is represented by two rules controlling calcium increase and decrease depending on the ATP level:

```
Cell{Ca_100:ca+ATP_1000000:atp} -> Cell{Ca_100+Ca_100+ATP_1000000}
 @@ if (Ca_min + Ca_diff/(1+exp(Ca_rise*(-1*(#atp)+Ca_ATP))))>
 #ca then k_ca_flux;
```
(S15)

```
Cell{Ca_100:ca+ATP_1000000:atp} -> Cell{ATP_1000000}
 @@ if (Ca_min + Ca_diff/(1+exp(Ca_rise*(-1*(#atp)+Ca_ATP))))<
 #ca then k_ca_flux;
```
(S16)

This set of rules is a simplified version of a more complex process. Rising ATP concentration triggers the closure of ATP-sensitive potassium (K^+) channels, leading to a depolarization of the cell membrane. Due to the reduced cell membrane potential, voltage-gated calcium (Ca^{2+}) channels open, allowing calcium to enter the cell. However, for this model and the timescale of minutes/hours, we used simple rules that couples the ATP level to a Ca^{2+} level.
Insulin secretion
The insulin secretion dynamics follow [24], where Ca^{2+} triggers the release of insulin from the rapid release pool (RRP).

```
Cell -> Cell{RRP_100} @@ if ##(Ca_100 in Cell)<Ca_threshold then
 kr_gluc*Cell.Gluc+kr_offset else
 kr_gluc*Cell.Gluc+kr_offset+kr_ca*(##(Ca_100 in Cell)-Ca_threshold);
```
(S17)

The glucose level in the cell controls RRP replenishment.

```
RRP_100:rrp -> Ins_100 @@ if ##(Ca_100 in Cell)<Ca_threshold then
 kf_ca1*(##(Ca_100 in Cell))*#rrp else
 kf_ca2*(##(Ca_100 in Cell))*#rrp;
```
(S18)

1.2.3 Initial conditions

Species	Number	Ref.
Cell(Gluc = 2.5[millimol/L])	1	-
Mito	75	[14]
pyruvat_1000000	0	-
ATP_1000000	4000	[25]
RRP_100	0	-
Ins_100	0	-
Ca_100	500	[19]

Table S5. Initialization of the ATP-Insulin secretion submodel.

1.2.4 Parameters

Parameter	Description	Value	Ref.
vol_cell	Volume of a Min6 cell	1025 $\mu^3 m^3$	[1]
NA	Avogadro constant	6.022 1/mol	-
Vm_glyc	Rate coefficient of glycolysis	3408.964 1/min	fit to [25, 26]
Km_glyc	Michaelis constant of glycolysis	4.626 mM	[25]
Ki_glyc	Inhibition constant of glycolysis	42806.181	fit to [25, 26]
Vmax_LDH	Rate coefficient of LDH	5450 [1/min]	[27]
Km_LDH_pyr	Michaelis constant of LDH	47.5 [μM]$*$vol_cell $*$ NA	[28]
Vmax_PDH	Rate coefficient of oxPhos	363.33 [1/min]	[26, 27]
Km_PDH_pyr	Michaelis constant of oxPhos	47.5 [μM]$*$vol_cell $*$ NA	[28]
k_ATP_u	Rate coefficient of ATP use	0.748 [1/min]	fit to [25, 26]
Ca_min	Rate parameter of Ca^{2+} flux	486.4	[19]
Ca_diff	Rate parameter of Ca^{2+} flux	742.5	[19]
Ca_rise	Rate parameter of Ca^{2+} flux	0.759*10^{-3}	[19]
Ca_ATP	Rate parameter of Ca^{2+} flux	8719	[19]
k_ca_flux	Rate parameter of Ca^{2+} flux	10 [1/s]	-
Ca_threshold	Ca^{2+} act. threshold of GSIS	852.936	fit to [29]
kr_gluc	Rate parameter of GSIS	261.542 [1/Ms]	fit to [29]
kr_offset	Rate parameter of GSIS	4.796 [Hz]	fit to [29]
kr_ca	Rate parameter of GSIS	0.103 [1/s]	fit to [29]
kf_ca1	Rate parameter of GSIS	0.125*10^{-2} [1/s]	fit to [29]
kf_ca2	Rate parameter of GSIS	0.326*10^{-2} [1/s]	fit to [29]

Table S6. Parameters used in the submodel.

1.2.5 Parameter Estimation

The submodel was used to estimate the ATP and insulin secretion dynamic parameters in three steps, as shown in the provenance graph in Fig.3 of the main paper. First, only the initial four rules describing ATP generation were used to fit the parameters Vm_glyc, Ki_glyc, and k_ATP_u to the glucose-dependent ATP production observed in Min6 cells. As fitting targets, both the intracellular ATP content at different glucose concentrations [25] and ATP production rates derived from the oxygen consumption rate (OCR) and extracellular acidification rate (ECAR) were used. The latter were

calculated from the data reported by [26] using the equations provided in [30]. Parameter optimization was performed using pyABC, minimizing the relative quadratic error. This method gives back the posterior distribution of the parameters that minimize the error. The advantage of having a posterior distribution instead of just the parameter value that minimizes the error is that additional information can be extracted. From the shape of the posterior distribution, it can be seen whether the parameter converges, whether the output is sensitive to the parameter, and whether multiple plausible parameter values exist. The downside of the method is the large number of replications needed to estimate the posterior distribution, limiting its use to simulations with short runtimes. As an output, pyABC generates figures as shown in Fig. S7 that include the marginal distributions of the parameters in the diagonal, the kernel density estimation on the lower diagonal, and scatter plots of the weighted particles on the top diagonal. From the shape of the marginal distribution, it can be seen if the parameter estimation has converged or if the output is insensitive to the parameter. In the density estimates and scatter plots, relationships between the parameters are evident.

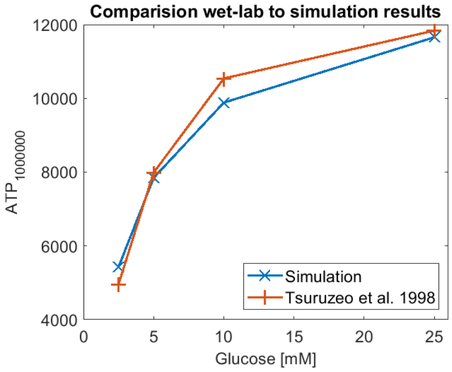

Fig. S6. Comparison of ATP levels reported by [25] and the simulation results.

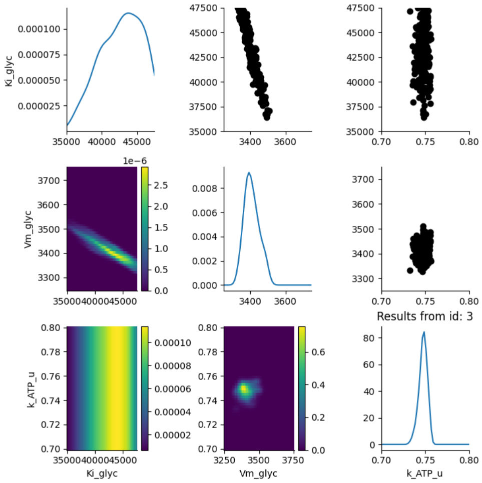

Fig. S7. Output of pyABC with marginal distributions on the diagonal, scatter plots of the weighted particles on the upper diagonal, and kernel density estimated on the lower diagonal.

The simulated ATP data show good agreement with the experimental measurements across different glucose levels (Fig. S6). Fig. S7 shows the posterior distributions of the three fitted parameters, all of which show convergence. After fitting the ATP dynamics, the calcium dynamics were added. The parameters `Ca_min`, `Ca_diff`, `Ca_rise`, and `Ca_ATP` from the rules S15 and S16 were estimated based on the calcium levels at different glucose levels reported in [19]. Finally, the parameters of the insulin secretion dynamics were estimated. The parameters in question were `Ca_threshold`, `kr_gluc`, `kr_offset`, `kr_ca`, `kf_ca1`, and `kf_ca2`. Parameter estimation was again performed using pyABC (with a relative quadratic error) based on the glucose-dependent insulin secretion data for Min6 cells [29].

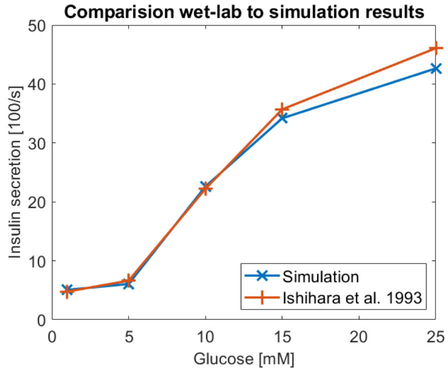

Fig. S8. Comparison of the insulin secretion reported by [29] and the simulation model.

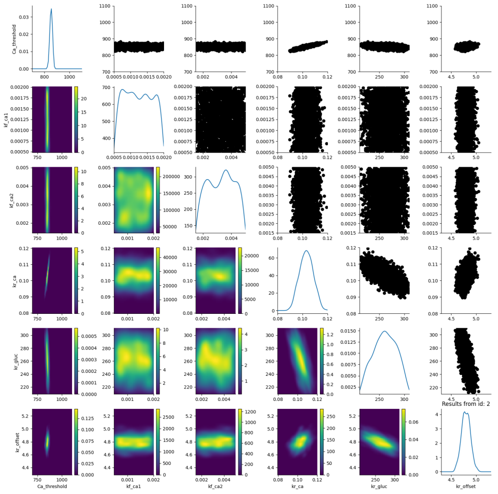

Fig. S9. Output of pyABC with marginal distributions on the diagonal, scatter plots of the weighted particles on the upper diagonal, and kernel density estimated on the lower diagonal.

The simulated insulin secretion dynamics show good agreement with experimental data (Fig. S8). Fig. S9 illustrates the posterior distributions of the six fitted parameters. It is notable that the distribution of the parameters `kf_ca1` and `kf_ca2` does not converge. These parameters are linked to the triggering pathway of insulin secretion but are insensitive to long-term behavior. Since the insulin secretion data from [29] (see fig. S8) only contains the accumulated insulin secretion over 2 hours and no time-resolved insulin secretion, these parameters have little effect on the simulation output and can not be determined by pyABC. To estimate these parameters, time-resolved data of the initial insulin secretion dynamics would be needed. Addressing this short-term dynamic, however, lies beyond the scope of the present study.

1.3 cAMP model

The cAMP submodel focuses on the dynamics of cyclic AMP (cAMP) generation and degradation shown in Fig. S10. This submodel uses parts of the previous model, as discussed below.

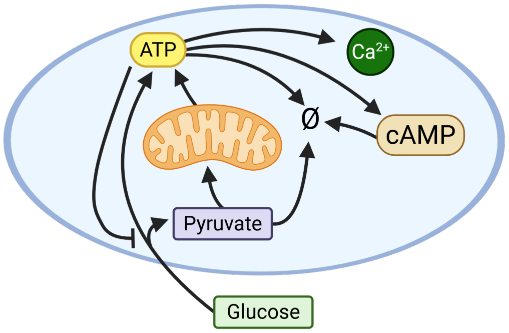

Fig. S10. Schematic representation of the cAMP model. Glucose is metabolized to ATP, which is turned into cAMP by adenylyl cyclase (AC). cAMP decays over time. Created in BioRender. Henning, P. (2026) `https://BioRender.com/xooeky5`

1.3.1 Species

Species	Description
Cell(Gluc: float[mol/L])	beta-cell, Gluc: glucose level
Mito	Mitochondrion
pyruvat_1000000	10^6 pyruvate molecules
ATP_1000000	10^6 ATP molecules
Ca_100	100 Ca^{2+} atomes
cAMP_100	100 cAMP molecules

Table S7. Species used in the cAMP model.

1.3.2 Rules

The cAMP-submodel includes the first six rules (rules S11, S12, S13, S14, S15, and S16) of the previous model. Additionally, it includes a rule to model the generation of cAMP through adenylyl cyclase (AC) [31] and its subsequent degradation.

cAMP generation

As in other models [32], the rate of cAMP generation depends only on the intracellular calcium level [33, 34], not on the ATP level. This assumption is supported by the fact that the $K_{m,ATP}$ value of AC (0.11 - 0.34 mM) [35, 36] is much lower than the physiological ATP concentration in pancreatic beta cells (approximately 6–18 mM) [25]. The rate expression is derived from experimental data on AC activity at varying calcium levels [37].

```
Cell -> Cell{cAMP_100}
  @@ vmax_AC*((##(Ca_100 in Cell))^2)/(Km_Ca^2+((##(Ca_100 in Cell))^2));
```
(S19)

cAMP decay

The degradation of cAMP is modeled as a first-order decay process with a constant rate [38, 39].

```
Cell{cAMP_100} -> Cell @ k_cAMP_d;
```
(S20)

1.3.3 Initial conditions

Species	Number	Ref.
Cell(Gluc = 2[millimol/L])	1	-
Mito()	75	[14]
pyruvat_1000000	0	-
ATP_1000000	4000	[25]
Ca_100	500	[19]
cAMP_100	6200	[18]

Table S8. Initialization of the cAMP submodel.

1.3.4 Parameters

Parameter	Description	Value	Ref.
vol_cell	Volume of a Min6 cell	1025 $\mu^3 m^3$	[1]
NA	Avogadro constant	6.022 1/mol	-
Vm_glyc	Rate coefficient of glycolysis	3408.964 1/min	see tab. S6
Km_glyc	Michaelis constant of glycolysis	4.626 mM	[25]
Ki_glyc	Inhibition constant of glycolysis	42806.181	see tab. S6
Vmax_LDH	Rate coefficient of LDH	5450 [1/min]	[27]
Km_LDH_pyr	Michaelis constant of LDH	47.5 [μM]$*$vol_cell $*$ NA	[28]
Vmax_PDH	Rate coefficient of oxPhos	363.33 [1/min]	[26, 27]
Km_PDH_pyr	Michaelis constant of oxPhos	47.5 [μM]$*$vol_cell $*$ NA	[28]
k_ATP_u	Rate coefficient of ATP use	0.748 [1/min]	see tab. S6
Ca_min	Rate parameter of Ca^{2+} flux	486.4	[19]
Ca_diff	Rate parameter of Ca^{2+} flux	742.5	[19]
Ca_rise	Rate parameter of Ca^{2+} flux	0.759*10^{-3}	[19]
Ca_ATP	Rate parameter of Ca^{2+} flux	8719	[19]
k_ca_flux	Rate parameter of Ca^{2+} flux	10 [1/s]	-
vmax_AC	Rate coefficient of AC	8000 [1/min]	-
k_cAMP_d	Decay of cAMP	0.2602 [1/min]	fit to [18]
Km_Ca	Activation of AC	1045	fit to [18]

Table S9. Parameters used in the submodel.

The parameter vmax_AC was set arbitrarily since the final level of cAMP depends on the ratio between the creation and decay of cAMP. Therefore, either vmax_AC or k_cAMP_d can be fixed to a sufficiently large value for cAMP to reach its steady state fast, and the other value can be used as a fit parameter to determine the steady state.

1.3.5 Parameter Estimation

The cAMP submodel was used to estimate the parameters k_cAMP_d and Km_Ca, which describe cAMP decay and calcium-dependent activation, respectively. Experimental data from [18] were used as reference. Because quantitative cAMP measurements at multiple glucose levels in beta cells are scarce, the dataset includes cAMP concentrations at only two glucose concentrations. Parameter estimation was performed using pyABC, minimizing the relative quadratic error.

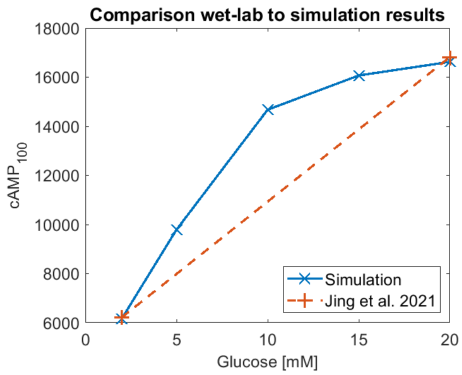

Fig. S11. Comparison of the cAMP levels reported by [18] and simulation results from the cAMP submodel.

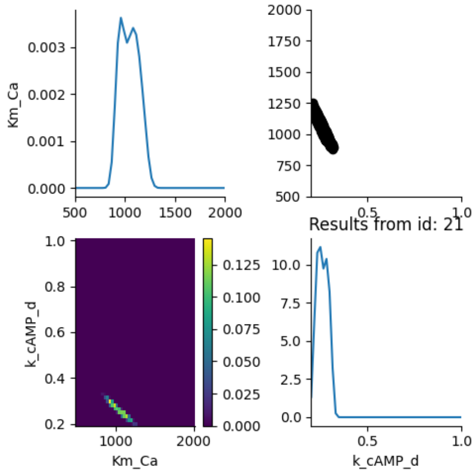

Fig. S12. Output of pyABC with marginal distributions on the diagonal, scatter plots of the weighted particles on the upper diagonal, and kernel density estimated on the lower diagonal.

Fig. S11 shows that the simulated cAMP levels are in good agreement with the experimental data. The narrow posterior peaks in Fig. S12 indicate that both parameters are well constrained and that the distributions have converged.

2 Beta-Cell Model

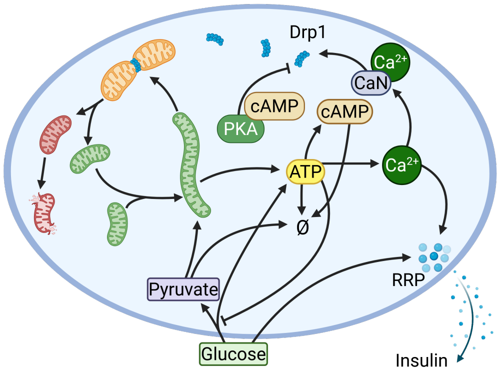

Fig. S13. Schematic representation of the complete beta-cell model, integrating the ATP–insulin secretion, cAMP, and mitochondrial fission–fusion submodels. Created in BioRender. Henning, P. (2026) `https://BioRender.com/s00y554`

For the complete beta cell model (see Fig. S13), all three submodels were combined, and some extensions and modifications were added. Specifically, the anchor protein used in the fission-fusion submodel was replaced by the two distinct anchor proteins, MFF and Fis1. Additionally, mitochondrial compartments were extended to include the attributes `health` and `damage`, while the species `Mito_vol` used in the original fission–fusion model was converted into an attribute of each mitochondrion. For each 0.01 μm^3 of mitochondrial volume, one unit of `health` or `damage` is assigned to a mitochondrion. So in a mitochondrion with a size of 1 μm^3, a typicall size in Min6 cells [14], the health

and damage attribute would add up to 100. To ensure that this ratio remains constant, it was necessary to model the volume as an attribute as well.

2.1 Species

Species	Description
Cell(Gluc: float[mol/L], mito_vol:int*)	beta-cell, Gluc: glucose level, mito_vol: volume of all mitochondria
Mito(vol:int*, health:int*, damage:int*, rand1:float*, rand2:float*)	Mitochondrion, vol: volume, health: health, damage: damage rand1/2: used for fission
Drp1_10(p:bool)	10 Drp1 proteins, p: phosphorization state
Fis1	Fis1 molecule
MFF	MFF molecule
PKA	cAMP-dependent protein kinase
cAMP	cyclic AMP
CaN	Calcineurin
Ca_100	100 Ca^{2+}
pyruvat_1000000	10^6 pyruvate molecules
ATP_1000000	10^6 ATP molecules
RRP_100	100 insulin in rapid releasable pool (RRP)
Ins_100	100 insulin molecules

Table S10. Species used in the beta-cell model.

2.2 Rules

Drp1 binding
Besides the changes from a general anchor protein to MFF and Fis1, the rules for Drp1 binding remain the same as rule S1.

```
Mito{Fis1} + Drp1_10(p==false) -> Mito{Fis1{Drp1_10}} @ kon/vol_cell;
Mito{MFF} + Drp1_10(p==false) -> Mito{MFF{Drp1_10}} @ kon/vol_cell;
```
(S21)

Drp1 unbinding
The unbinding rules remain the same as in rule S2, besides the change of the anchor proteins.

```
Mito{Fis1:f{Drp1_10}} -> Mito{Fis1} + Drp1_10 @@ 1/10*koff*#f;
Mito{MFF:m{Drp1_10}} -> Mito{MFF} + Drp1_10 @@ 1/10*koff*#m;
```
(S22)

Drp1 (de)phosphorylation
The rules for the phosphorization of Drp1 by PKA (rule S3) and the dephosphorization by CaN (rule S4) remain unchanged.
Mitochondrial fission
The fission rules were extended by the redistribution of the health and damage attributes. For the midzone fission, health and fission are divided proportionally to the size of the new sister mitochondria.

```
Mito:m{MFF{Drp1_10:drp + ?solA}} ->
 Mito(rand1 = RandomGauss(0.5, 0.075)){MFF} + Drp1_10+?solA
 @@ if (#drp>N_fission) then k_fission*(#(Mito_vol in m))^3;
```
(S23)

```
Mito(rand1<1000):m{?sol1[Mito.rand1] + ?sol2[1-Mito.rand1]} ->
 Mito(vol=ceil(m.vol*m.rand1), health=ceil(m.health*m.rand1),
 damage=ceil(m.damage*m.rand1), rand1=1001, rand2=1001){?sol1} +
 Mito(vol=floor(m.vol*(1-m.rand1)), health = floor(m.health*(1-m.rand1)),
 damage = floor(m.damage*(1-m.rand1)), rand1=1001, rand2=1001){?sol2}
 @@ immediately;
```
(S24)

In the peripheral fission event, the smaller mitochondria receive a disproportionately high amount of damage, controlled by the parameter asym_fission.

```
Mito:m{Fis1{Drp1_10:drp + ?solA}} ->
 Mito(rand2 = RandomGauss(0.2, 0.04)){Fis1}+Drp1_10+?solA
 @@ if (#drp>N_fission) then k_fission*(m.vol)^3;
```
(S25)

```
Mito(rand2<1000, rand2>asym_fission, health+damage>=2):m
{?sol1[Mito.rand2] + ?sol2[1-Mito.rand2]} ->
 Mito(vol=ceil(m.vol*m.rand2), health=ceil(m.health*m.rand2-
 m.damage*asym_fission), damage=ceil(m.damage*(m.rand2+asym_fission)),
 rand1=1001, rand2=1001){?sol1} +
 Mito(vol=floor(m.vol*(1-m.rand2)), health=floor(m.health*(1-m.rand2)+
 m.damage*asym_fission), damage=floor(m.damage*((1-m.rand2)-asym_fission)),
 rand1=1001, rand2=1001){?sol2} @@ immediately;
```
(S26)

In addition, a rule was added to the peripheral fission that prevents the creation of mitochondria with negative health.

```
Mito(rand2<=asym_fission) -> Mito(rand2=asym_fission + 0.0001)
@@ immediately;
```
(S27)

Mitochondrial Fusion
In a fusion event, the health, damage, volume, and content of the two fusion mitochondria are combined in newly formed mitochondrion. This reaction can only occur, if the damage of the mitochondria is below the threshold Thr_damage [40].

```
Mito:m1{?solm1} + Mito:m2{?solm2} ->
 Mito(vol=m1.vol+m2.vol, health=m1.health+m2.health,
 damage=m1.damage+m2.damage, rand1=1001, rand2=1001){?solm1 + ?solm2}
 @@ if ((m1.damage/(m1.health+m1.damage))<Thr_damage &&
 (m2.damage/(m2.health+m2.damage))<Thr_damage) then k_fusion;
```
(S28)

ATP dynamic
For the ATP generate the rules S11, S12, S14 remain the same. The reaction rate of oxidative phosphorylation is modified by a factor that considers that mitochondria with low health are less active [41–46]. See also eq. 5 of the main text.

```
Mito:m + pyruvat_1000000:pyr -> Mito + 15 ATP_1000000 @@
 f_ATP*(0.1+0.9/(1+exp(damage_m*(damage_lim-m.health/(m.health+
 m.damage)))))*(m.vol/N0)*Vmax_PDH*#pyr*1e6/(#pyr*1e6+Km_PDH_pyr);
```
(S29)

Ca^{2+}, cAMP, and insulin dynamic

The rules for the Ca^{2+} dynamic (rules S15 and S16), the cAMP dynamics (rules S19 and S20), and the insulin secretion (rules S17 and S18) are the same as in the submodel from section 1.2.

Mitochondrial damage

The primary addition in the comprehensive beta cell model is the representation of damage dynamics. It consists of the damage dealt to mitochondria during their metabolic activity [47].

```
Mito(health>0):m + pyruvat_1000000:pyr ->
 Mito(damage = m.damage+1, health = m.health-1) + pyruvat_1000000 @@
 f_ATP*(1+0.5*m.damage/(m.health+m.damage))*(0.1+0.9/(1+exp(damage_m*
 (damage_lim-m.health/(m.health+m.damage)))))*(m.vol/N0)*0.007*
 Vmax_PDH*#pyr*1e6/(#pyr*1e6+Km_PDH_pyr);
```

(S30)

Mitochondrial repair

Parts of this damage can be repaired by the turnover of proteins [48–50] or mtDNA [41, 48].

```
Mito(damage>0):m -> Mito(damage = m.damage-1, health = m.health+1)
 @@ k_repair*m.damage;
```

(S31)

Mitophagy

The repair rate is not sufficient to compensate for all the damage caused by the oxPhos. Therefore, damage accumulates over time in the mitochondrial network. This damage is removed by the removal of (sufficiently small) mitochondria with a large amount of damage [50].

```
Cell:c{Mito:m{?solm}} -> Cell(mito_vol=c.mito_vol-m.vol){?solm}
 @@ if (m.vol < 50 && m.damage/(m.damage+m.health) > Thr_damage)
 then 10 [Hz];
```

(S32)

If a mitochondrion is removed, the MFF and Fis1 anchor sites bind to a new mitochondrion, holding the amount of Fis1 and MFF anchor sites constant.

```
Cell{Mito:m + Fis1} -> Cell{Mito{Fis1}} @@ 1000 [Hz]*(m.vol);
Cell{Mito:m + MFF} -> Cell{Mito{MFF}} @@ 1000 [Hz]*(m.vol);
```

(S33)

Biogenesis

To compensate for the loss of mitochondrial mass through mitophagy, a biogenesis rule is introduced that enables mitochondria to grow.

```
Cell:c{Mito:m} -> Cell(mito_vol=c.mito_vol+1){Mito(vol = m.vol+1,}
 health = m.health+1) @@ k_biog*N0/(1+exp((c.mito_vol-(N0-100))/gamma));
```

(S34)

2.3 Initial conditions

Species	Number	Ref.
Cell(Gluc = 1[milli mol/L], mito_vol = 6800)	1	-
Mito(vol = 100, health = 70, damage = 30, rand1 = 1001, rand2 = 1001)	68	[14]
Drp1_10(p = true)	10000	[15]
Drp1_10(p = false)	10000	[15]
Fis1	306	[16, 17]
MFF	306	[16, 17]
PKA	300000	[15]
cAMP	6200	[18]
CaN	350000	[15]
Ca_100	500	[19]
pyruvat_1000000	0	-
ATP_1000000	4000	[25]
RRP_100	0	-
Ins_100	0	-

Table S11. Initialization of the beta-cell submodel.

2.4 Parameters

Parameter	Description	Value	Ref.
vol_cell	Volume of a Min6 cell	1025 μm^3	[1]
NA	Avogadro constant	6.022 1/mol	-
kon	Binding rate or Drp1	0.025 $\mu m^3/s$	fit to [5, 12, 14, 20]
Kd	Equi. constant for Drp1 binding	0.301 $\mu mol/s$	fit to [5, 12, 14, 20]
koff	Unbinding rate of Drp1	kon ∗ Kd	-
k_fission	Rate coefficient for fission	$8.211 \cdot 10^{-8} Hz$	fit to [5, 12, 14, 20]
N_fission	Number of Drp1 needed for fission	11	[5]
rand1	Position of midzone fission	N(0.5, 0.075)	[11]
rand2	Position of peripheral fission	N(0.2, 0.004)	[11]
asym_fission	Fission asymmetry	0-10%	-
k_fusion	Rate coefficient for fusion	$5.41 \cdot 10^{-4}$ 1/min	[12, 20]
kcatCaN	Rate constant for CaN	7.6 1/s	[9]
KMCaN	Michaelis constant for CaN	160 mM	[9]
K_act_CaN	Activation constant of CaN	14 nM	[10]
kcatpka	Rate constant ok PKA	4 1/s	[21]
KMpka	Michaelis constant for PKA	70 mM	[21]
K_act_PKA	Activation constant of PKA	0.9 μM	[7]
Vm_glyc	Rate coefficient of glycolysis	3408.964 1/min	fit to [25, 26]
Km_glyc	Michaelis constant of glycolysis	4.626 mM	[25]
Ki_glyc	Inhibition constant of glycolysis	42806.181	fit to [25, 26]
Vmax_LDH	Rate coefficient of LDH	5450 [1/min]	[27]
Km_LDH_pyr	Michaelis constant of LDH	47.5 [μM]·vol_cell · NA	[28]
Vmax_PDH	Rate coefficient of oxPhos	363.33 [1/min]	[26, 27]
Km_PDH_pyr	Michaelis constant of oxPhos	47.5 [μM]·vol_cell · NA	[28]
f_ATP	Scaling factor for oxPhos	1.54	fit to [20, 29]
damage_m	Steepness of damage response	28.7	fit to [20, 29]
damage_lim	Point of damage response	53.2%	fit to [20, 29]
k_ATP_u	Rate coefficient of ATP use	0.748 [1/min]	fit to [25, 26]
Ca_min	Rate parameter of Ca^{2+} flux	486.4	[19]
Ca_diff	Rate parameter of Ca^{2+} flux	742.5	[19]
Ca_rise	Rate parameter of Ca^{2+} flux	$0.759 \cdot 10^{-3}$	[19]
Ca_ATP	Rate parameter of Ca^{2+} flux	8719	[19]
k_ca_flux	Rate parameter of Ca^{2+} flux	10 [1/s]	-
Ca_threshold	Ca^{2+} act. threshold of GSIS	852.936	fit to [29]
kr_gluc	Rate parameter of GSIS	261.542 [1/Ms]	fit to [29]
kr_offset	Rate parameter of GSIS	4.796 [Hz]	fit to [29]
kr_ca	Rate parameter of GSIS	0.103 [1/s]	fit to [29]
kf_ca1	Rate parameter of GSIS	$0.125 \cdot 10^{-2}$ [1/s]	fit to [29]
kf_ca2	Rate parameter of GSIS	$0.326 \cdot 10^{-2}$ [1/s]	fit to [29]
vmax_AC	Rate coefficient of AC	8000 [1/min]	-
k_cAMP_d	decay of cAMP	0.2602 [1/min]	fit to [18]
Km_Ca	Activation of AC	1045	fit to [18]
N0	Normal mitochondiral mass	6800	[14]
gamma	Parameter of biogenesis	15	-
k_biog	Rate parameter of biogenesis	0.05 [1/day]	[49–51]
k_repair	Rate or repair	0.1 [1/day]	[49–51]
Thr_damage	Threshold for fission and mitophagy	15-85%	-

Table S12. Parameters used in the beta cell model.

2.5 Parameter Estimation

The model was used to estimate parameters for the damage regulation. Details are described in the section *Interplay of Mitochondrial Damage and Insulin Secretion* of the main text.

In addition, we tested whether the complete beta cell model still reproduces the data used for parameter estimation in the submodels, to ensure that neither the interaction between submodel components nor the new features of the model alter the model's basic behaviour. For the simulation, the parameters from Table 1 of the main text were used.

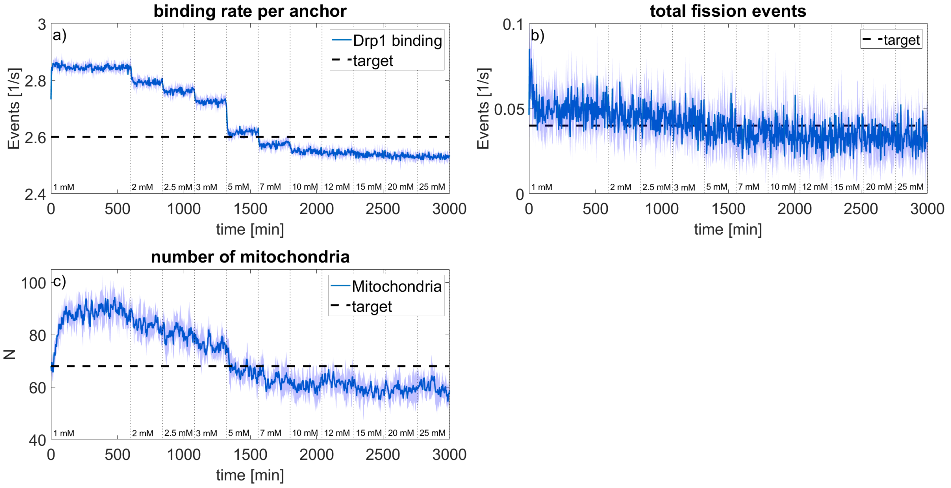

Fig. S14. Comparison between literature data and simulation results for a) the Drp1 binding rate [5], b) the mitochondrial fission rate [12, 20], and c) the number of mitochondria in a Min6 cell [14]. The shaded area shows the standard derivation of N=5 simulation runs and the numbers at the bottom note the glucose level.

The output of the fission fusion dynamic is shown in Fig. S14. Initially, the model runs for 10 hours at 1 mM glucose to reach its steady state. After the steady state was achieved, the glucose level was increased every 4 hours. All observed parameters change in relation to the glucose level. For the Drp1 binding rate simulation results are within the range of the reported rate (2.6±1.7 1/s [5]). The number of mitochondria also lies within the range reported in the literature (50-100 mitochondria [14]). The fission rate varies slightly more than the reported values(0.039±0.006 fusion/mitochondrion/min [12] and 0.0457 fusion/mitochondrion/min, no standard deviation reported [20]); however, in the wet-lab experiments, the glucose concentration was not changed.

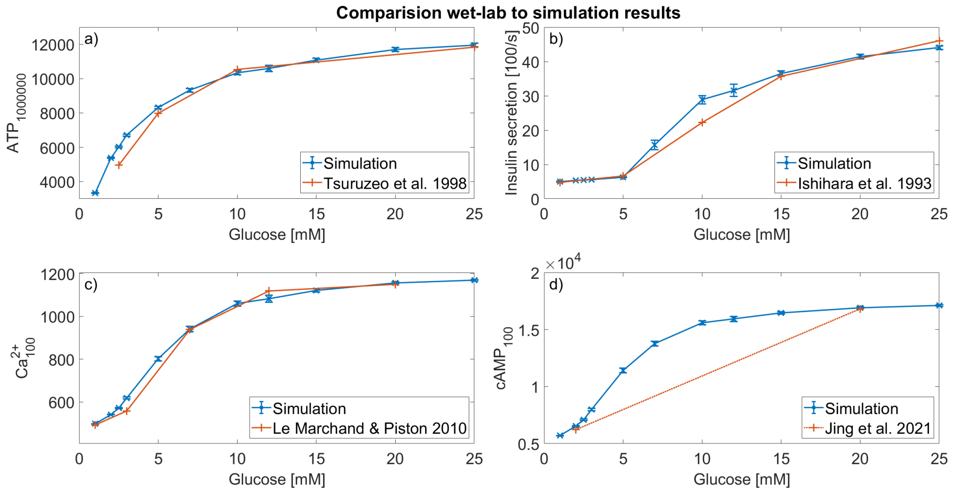

Fig. S15. Comparison between the experimental and simulated data for a) ATP concentration, b) insulin secretion, c) calcium concentration, and d) cAMP concentration for different glucose levels. N=5 replications.

The simulated ATP, calcium, and cAMP concentrations, as well as the insulin secretion shown in Fig. S15 are still in good agreement in the complete beta cell model.

References

1. A. Z. Higgins and J. O. M. Karlsson, "Curve fitting approach for measurement of cellular osmotic properties by the electrical sensing zone method. I. Osmotically inactive volume," *Cryobiology*, vol. 57, pp. 223–233, Dec. 2008.

2. Firdos and A. Mittal, "Secretory Conservation in Insulin Producing Cells: Is There a System-Level Law of Mass Action in Biology?," *ACS Omega*, vol. 8, pp. 37573–37583, Oct. 2023. Publisher: American Chemical Society.

3. J. T. Cribbs and S. Strack, "Reversible phosphorylation of Drp1 by cyclic AMP-dependent protein kinase and calcineurin regulates mitochondrial fission and cell death," *EMBO reports*, vol. 8, pp. 939–944, Oct. 2007. Publisher: John Wiley & Sons, Ltd.

4. A. K. Leinheiser, C. C. Mitchell, E. Rooke, S. Strack, and C. E. Grueter, "A dynamical systems model for the total fission rate in Drp1-dependent mitochondrial fission," *PLOS Computational Biology*, vol. 20, p. e1012596, Nov. 2024. Publisher: Public Library of Science.

5. B. M. Michalska, K. Kwapiszewska, J. Szczepanowska, T. Kalwarczyk, P. Patalas-Krawczyk, K. Szczepański, R. Hołyst, J. Duszyński, and J. Szymański, "Insight into the fission mechanism by quantitative characterization of Drp1 protein distribution in the living cell," *Scientific Reports*, vol. 8, p. 8122, May 2018. Number: 1 Publisher: Nature Publishing Group.

6. K. A. Nolden, M. C. Harwig, and R. B. Hill, "Human Fis1 directly interacts with Drp1 in an evolutionarily conserved manner to promote mitochondrial fission," *Journal of Biological Chemistry*, vol. 299, Dec. 2023. Publisher: Elsevier.

7. D. Vigil, J.-H. Lin, C. A. Sotriffer, J. K. Pennypacker, J. A. McCammon, and S. S. Taylor, "A simple electrostatic switch important in the activation of type I protein kinase A by cyclic AMP," *Protein Science*, vol. 15, no. 1, pp. 113–121, 2006. _eprint: https://onlinelibrary.wiley.com/doi/pdf/10.1110/ps.051723606.

8. M. M. Vetter, H.-M. Zenn, E. Méndez, H. van den Boom, F. W. Herberg, and B. S. Skålhegg, "The testis-specific Cα2 subunit of PKA is kinetically indistinguishable from the common Cα1 subunit of PKA," *BMC Biochemistry*, vol. 12, p. 40, Aug. 2011.

9. A. M. Slupe, R. A. Merrill, K. H. Flippo, M. A. Lobas, J. C. D. Houtman, and S. Strack, "A Calcineurin Docking Motif (LXVP) in Dynamin-related Protein 1 Contributes to Mitochondrial Fragmentation and Ischemic Neuronal Injury *," *Journal of Biological Chemistry*, vol. 288, pp. 12353–12365, Apr. 2013. Publisher: Elsevier.

10. A. R. Quintana, D. Wang, J. E. Forbes, and M. N. Waxham, "Kinetics of calmodulin binding to calcineurin," *Biochemical and Biophysical Research Communications*, vol. 334, pp. 674–680, Aug. 2005.

11. T. Kleele, T. Rey, J. Winter, S. Zaganelli, D. Mahecic, H. Perreten Lambert, F. P. Ruberto, M. Nemir, T. Wai, T. Pedrazzini, and S. Manley, "Distinct fission signatures predict mitochondrial degradation or biogenesis," *Nature*, vol. 593, pp. 435–439, May 2021. Number: 7859 Publisher: Nature Publishing Group.

12. M. Cagalinec, D. Safiulina, M. Liiv, J. Liiv, V. Choubey, P. Wareski, V. Veksler, and A. Kaasik, "Principles of the mitochondrial fusion and fission cycle in neurons," *Journal of Cell Science*, vol. 126, pp. 2187–2197, May 2013.

13. Z. Y. Tam, J. Gruber, B. Halliwell, and R. Gunawan, "Mathematical Modeling of the Role of Mitochondrial Fusion and Fission in Mitochondrial DNA Maintenance," *PLOS ONE*, vol. 8, p. e76230, Oct. 2013. Publisher: Public Library of Science.

14. A. Chaudhry, R. Shi, and D. S. Luciani, "A pipeline for multidimensional confocal analysis of mitochondrial morphology, function, and dynamics in pancreatic β-cells," *American Journal of Physiology-Endocrinology and Metabolism*, vol. 318, pp. E87–E101, Feb. 2020. Publisher: American Physiological Society.

15. F. Sacco, S. J. Humphrey, J. Cox, M. Mischnik, A. Schulte, T. Klabunde, M. Schäfer, and M. Mann, "Glucose-regulated and drug-perturbed phosphoproteome reveals molecular mechanisms controlling insulin secretion," *Nature Communications*, vol. 7, p. 13250, Nov. 2016. Number: 1 Publisher: Nature Publishing Group.

16. A. S. Moore, Y. C. Wong, C. L. Simpson, and E. L. F. Holzbaur, "Dynamic actin cycling through mitochondrial subpopulations locally regulates the fission–fusion balance within mitochondrial networks," *Nature Communications*, vol. 7, p. 12886, Sept. 2016. Number: 1 Publisher: Nature Publishing Group.

17. F. Montecinos-Franjola, B. L. Bauer, J. A. Mears, and R. Ramachandran, "GFP fluorescence tagging alters dynamin-related protein 1 oligomerization dynamics and creates disassembly-refractory puncta to mediate mitochondrial fission," *Scientific Reports*, vol. 10, p. 14777, Sept. 2020. Number: 1 Publisher: Nature Publishing Group.

18. M. Jing, S. Wang, D. Li, Z. Wang, Z. Li, Y. Lu, T. Sun, C. Qiu, F. Chen, H. Yu, and W. Zhang, "Lorcaserin Inhibit Glucose-Stimulated Insulin Secretion and Calcium Influx in Murine Pancreatic Islets," *Frontiers in Pharmacology*, vol. 12, 2021.

19. S. J. Le Marchand and D. W. Piston, "Glucose Suppression of Glucagon Secretion: METABOLIC AND CALCIUM RESPONSES FROM α-CELLS IN INTACT MOUSE PANCREATIC ISLETS," *Journal of Biological Chemistry*, vol. 285, pp. 14389–14398, May 2010.

20. G. Twig, A. Elorza, A. J. A. Molina, H. Mohamed, J. D. Wikstrom, G. Walzer, L. Stiles, S. E. Haigh, S. Katz, G. Las, J. Alroy, M. Wu, B. F. Py, J. Yuan, J. T. Deeney, B. E. Corkey, and O. S. Shirihai, "Fission and selective fusion govern mitochondrial segregation and elimination by autophagy," *The EMBO Journal*, vol. 27, pp. 433–446, Jan. 2008. Num Pages: 446 Publisher: John Wiley & Sons, Ltd.

21. "Information on EC 2.7.11.11 - cAMP-dependent protein kinase - BRENDA Enzyme Database."

22. L. J. V. Miranda, "PySwarms, a research-toolkit for Particle Swarm Optimization in Python," *Journal of Open Source Software*, vol. 3, 2018.

23. N. S. Chandel, "Glycolysis," *Cold Spring Harbor Perspectives in Biology*, vol. 13, p. a040535, May 2021. Company: Cold Spring Harbor Laboratory Press Distributor: Cold Spring Harbor Laboratory Press Institution: Cold Spring Harbor Laboratory Press Label: Cold Spring Harbor Laboratory Press Publisher: Cold Spring Harbor Lab.

24. E. Grespan, T. Giorgino, S. Arslanian, A. Natali, E. Ferrannini, and A. Mari, "Defective Amplifying Pathway of β-Cell Secretory Response to Glucose in Type 2 Diabetes: Integrated Modeling of In Vitro and In Vivo Evidence," *Diabetes*, vol. 67, pp. 496–506, Dec. 2017.

25. K. Tsuruzoe, E. Araki, N. Furukawa, T. Shirotani, K. Matsumoto, K. Kaneko, H. Motoshima, K. Yoshizato, A. Shirakami, H. Kishikawa, J. Miyazaki, and M. Shichiri, "Creation and characterization of a mitochondrial DNA-depleted pancreatic beta-cell line: impaired insulin secretion induced by glucose, leucine, and sulfonylureas.," *Diabetes*, vol. 47, pp. 621–631, Apr. 1998.

26. X. Xiong, G. Wang, R. Tao, P. Wu, T. Kono, K. Li, W.-X. Ding, X. Tong, S. A. Tersey, R. A. Harris, R. G. Mirmira, C. Evans-Molina, and X. C. Dong, "Sirtuin 6 regulates glucose-stimulated insulin secretion in mouse pancreatic beta cells," *Diabetologia*, vol. 59, pp. 151–160, Jan. 2016.

27. L. Kong, Q. Zhao, X. Jiang, J. Hu, Q. Jiang, L. Sheng, X. Peng, S. Wang, Y. Chen, Y. Wan, S. Hou, X. Liu, C. Ma, Y. Li, L. Quan, L. Chen, B. Cui, and P. Li, "Trimethylamine N-oxide impairs β-cell function and glucose tolerance," *Nature Communications*, vol. 15, p. 2526, Mar. 2024. Publisher: Nature Publishing Group.

28. L. E. Fridlyand and L. H. Philipson, "Glucose sensing in the pancreatic beta cell: a computational systems analysis," *Theoretical Biology and Medical Modelling*, vol. 7, p. 15, May 2010.

29. H. Ishihara, T. Asano, K. Tsukuda, H. Katagiri, K. Inukai, M. Anai, M. Kikuchi, Y. Yazaki, J. I. Miyazaki, and Y. Oka, "Pancreatic beta cell line MIN6 exhibits characteristics of glucose metabolism and glucose-stimulated insulin secretion similar to those of normal islets," *Diabetologia*, vol. 36, pp. 1139–1145, Nov. 1993.

30. S. A. Mookerjee, A. A. Gerencser, D. G. Nicholls, and M. D. Brand, "Quantifying intracellular rates of glycolytic and oxidative ATP production and consumption using extracellular flux measurements," *Journal of Biological Chemistry*, vol. 292, pp. 7189–7207, Apr. 2017. Publisher: Elsevier.

31. T. Kitaguchi, M. Oya, Y. Wada, T. Tsuboi, and A. Miyawaki, "Extracellular calcium influx activates adenylate cyclase 1 and potentiates insulin secretion in MIN6 cells," *Biochemical Journal*, vol. 450, pp. 365–373, Feb. 2013.

32. L. E. Fridlyand, M. C. Harbeck, M. W. Roe, and L. H. Philipson, "Regulation of cAMP dynamics by Ca2+ and G protein-coupled receptors in the pancreatic β-cell: a computational approach," *American Journal of Physiology-Cell Physiology*, vol. 293, pp. C1924–C1933, Dec. 2007. Publisher: American Physiological Society.

33. T. N. Litvin, M. Kamenetsky, A. Zarifyan, J. Buck, and L. R. Levin, "Kinetic Properties of "Soluble" Adenylyl Cyclase: SYNERGISM BETWEEN CALCIUM AND BICARBONATE*," *Journal of Biological Chemistry*, vol. 278, pp. 15922–15926, May 2003.

34. J. H. Zippin, Y. Chen, S. G. Straub, K. C. Hess, A. Diaz, D. Lee, P. Tso, G. G. Holz, G. W. G. Sharp, L. R. Levin, and J. Buck, "CO_2/HCO_3^-- and Calcium-regulated Soluble Adenylyl Cyclase as a Physiological ATP Sensor*," *Journal of Biological Chemistry*, vol. 288, pp. 33283–33291, Nov. 2013.

35. C. W. Dessauer and A. G. Gilman, "The Catalytic Mechanism of Mammalian Adenylyl Cyclase," *Journal of Biological Chemistry*, vol. 272, pp. 27787–27795, Oct. 1997.

36. A. Gille, G. H. Lushington, T.-C. Mou, M. B. Doughty, R. A. Johnson, and R. Seifert, "Differential Inhibition of Adenylyl Cyclase Isoforms and Soluble Guanylyl Cyclase by Purine and Pyrimidine Nucleotides," *Journal of Biological Chemistry*, vol. 279, pp. 19955–19969, May 2004.

37. Z. Wu, S. T. Wong, and D. R. Storms, "Modification of the calcium and calmodulin sensitivity of the type I adenylyl cyclase by mutagenesis of its calmodulin binding domain.," *Journal of Biological Chemistry*, vol. 268, pp. 23766–23768, Nov. 1993.

38. T. Issekutz, "Estimation of cyclic AMP turnover in normal and methylprednisolone-treated dogs: effect of catecholamines," *American Journal of Physiology-Legacy Content*, vol. 229, pp. 291–297, Aug. 1975. Publisher: American Physiological Society.

39. E. Gorin and S. Dickbuch, "Influence of epinephrine on the degradation of cyclic AMP in chicken erythrocytes," *Biochemical and Biophysical Research Communications*, vol. 91, pp. 1314–1320, Dec. 1979.

40. G. Twig, X. Liu, M. Liesa, J. D. Wikstrom, A. J. A. Molina, G. Las, G. Yaniv, G. Hajnóczky, and O. S. Shirihai, "Biophysical properties of mitochondrial fusion events in pancreatic β-cells and cardiac cells unravel potential control mechanisms of its selectivity," *American Journal of Physiology-Cell Physiology*, vol. 299, pp. C477–C487, Aug. 2010. Publisher: American Physiological Society.

41. F. M. Yakes and B. Van Houten, “Mitochondrial DNA damage is more extensive and persists longer than nuclear DNA damage in human cells following oxidative stress,” *Proceedings of the National Academy of Sciences*, vol. 94, pp. 514–519, Jan. 1997. Publisher: Proceedings of the National Academy of Sciences.

42. A. M. Furda, A. M. Marrangoni, A. Lokshin, and B. Van Houten, “Oxidants and not alkylating agents induce rapid mtDNA loss and mitochondrial dysfunction,” *DNA Repair*, vol. 11, pp. 684–692, Aug. 2012.

43. A. Sanz, P. Caro, J. Gómez, and G. Barja, “Testing the vicious cycle theory of mitochondrial ROS production: effects of H2O2 and cumene hydroperoxide treatment on heart mitochondria,” *Journal of Bioenergetics and Biomembranes*, vol. 38, pp. 121–127, Oct. 2006.

44. A. Cossu, A. M. Posadino, R. Giordo, C. Emanueli, A. M. Sanguinetti, A. Piscopo, M. Poiana, G. Capobianco, A. Piga, and G. Pintus, “Apricot Melanoidins Prevent Oxidative Endothelial Cell Death by Counteracting Mitochondrial Oxidation and Membrane Depolarization,” *PLOS ONE*, vol. 7, p. e48817, Nov. 2012. Publisher: Public Library of Science.

45. J. A. Armstrong, N. J. Cash, Y. Ouyang, J. C. Morton, M. Chvanov, D. Latawiec, M. Awais, A. V. Tepikin, R. Sutton, and D. N. Criddle, “Oxidative stress alters mitochondrial bioenergetics and modifies pancreatic cell death independently of cyclophilin D, resulting in an apoptosis-to-necrosis shift,” *Journal of Biological Chemistry*, vol. 293, pp. 8032–8047, May 2018. Publisher: Elsevier.

46. J. Fu, H. Zheng, H. Wang, B. Yang, R. Zhao, C. Lu, Z. Liu, Y. Hou, Y. Xu, Q. Zhang, W. Qu, and J. Pi, “Protective Role of Nuclear Factor E2-Related Factor 2 against Acute Oxidative Stress-Induced Pancreatic β-Cell Damage,” *Oxidative Medicine and Cellular Longevity*, vol. 2015, no. 1, p. 639191, 2015. _eprint: https://onlinelibrary.wiley.com/doi/pdf/10.1155/2015/639191.

47. M. P. Murphy, “How mitochondria produce reactive oxygen species,” *Biochemical Journal*, vol. 417, pp. 1–13, Dec. 2008.

48. R. M. Anson, D. L. Croteau, R. H. Stierum, C. Filburn, R. Parsell, and V. A. Bohr, “Homogenous repair of singlet oxygen-induced DNA damage in differentially transcribed regions and strands of human mitochondrial DNA,” *Nucleic Acids Research*, vol. 26, pp. 662–1997, Jan. 1998.

49. T.-Y. Kim, D. Wang, A. K. Kim, E. Lau, A. J. Lin, D. A. Liem, J. Zhang, N. C. Zong, M. P. Y. Lam, and P. Ping, “Metabolic Labeling Reveals Proteome Dynamics of Mouse Mitochondria *,” *Molecular & Cellular Proteomics*, vol. 11, pp. 1586–1594, Dec. 2012. Publisher: Elsevier.

50. E. S. Vincow, R. E. Thomas, G. E. Merrihew, N. J. Shulman, T. K. Bammler, J. W. MacDonald, M. J. MacCoss, and L. J. Pallanck, “Autophagy accounts for approximately one-third of mitochondrial protein turnover and is protein selective,” *Autophagy*, vol. 15, pp. 1592–1605, Sept. 2019. Publisher: Taylor & Francis _eprint: https://doi.org/10.1080/15548627.2019.1586258.

51. X. C. Y. Chan, C. M. Black, A. J. Lin, P. Ping, and E. Lau, “Mitochondrial protein turnover: Methods to measure turnover rates on a large scale,” *Journal of Molecular and Cellular Cardiology*, vol. 78, pp. 54–61, Jan. 2015.
